# Evidence-oriented teaching of geriatric psychiatry: a narrative literature synthesis and pilot evaluation of a clerkship seminar

**DOI:** 10.3205/zma001541

**Published:** 2022-04-14

**Authors:** Eric Lenouvel, Finn Lornsen, Brigitte Schüpbach, Janet Mattson, Stefan Klöppel, Severin Pinilla

**Affiliations:** 1 University of Bern, University Hospital of Old Age Psychiatry and Psychotherapy, Bern, Switzerland; 2 Karolinska Institute, LIME, Department of Learning, Informatics, Management and Ethics, Solna, Sweden; 3 University of Bern, Institute for Medical Education, Department for Assessment and Evaluation, Bern, Switzerland

**Keywords:** geriatric psychiatry, teaching, learning activities, undergraduate medical education

## Abstract

**Introduction:**

The field of geriatric psychiatry has in recent decades developed into an independent discipline, incorporating elements of psychiatry, neurology and internal medicine. In view of demographic changes, this field is becoming increasingly relevant for primary care and undergraduate medical training. So far, however, there is little educational guidance for instructional design of geriatric psychiatry in undergraduate medical education.

**Project description:**

A narrative literature review of medical education studies in the field of geriatric psychiatry was conducted. Student evaluations of a geriatric psychiatry clerkship seminar were analyzed, followed by a target group analysis. Results informed the iterative development of new clerkship seminar content and structure. This was implemented and evaluated over several academic cycles. Learning material was made available via the open-source learning management system “ILIAS”.

**Results:**

A total of 29 medical education articles were identified and evaluated. The previous seminar in geriatric psychiatry at our university hospital was rated below average (Likert item overall rating of 4.3/6 compared to other seminars with an average overall rating of 5.2, p<0.001). An evidence-oriented revision of the content and instructional design was implemented. Activation of learners, self-reference effect, and audience questioning were used during the lecture. Additionally, two geriatric psychiatry case scenarios were adapted for discussion. We saw continuous improvement of student evaluations of the revised course, reaching a rating improvement of 5.3 out of 6 (p<0.01, U=135.5 Cohen’s d=1.28).

**Conclusion:**

A systematic approach was used to develop a geriatric psychiatry clerkship seminar, based on medical education evidence, for undergraduate medical students, resulting in better student evaluations. The teaching materials can be adapted for local implementation at other teaching hospitals. Future studies should also explore effects regarding higher learning outcomes.

## Zusammenfassung

**Einleitung:**

Das Fachgebiet Alterspsychiatrie und Psychotherapie hat sich in den letzten Jahrzehnten zu einer eigenständigen Disziplin im Querschnittsgebiet Psychiatrie, Neurologie und Innere Medizin entwickelt. Angesichts der demographischen Entwicklungen gewinnt dieses auch an Bedeutung für die Grundversorgung und medizinische Ausbildung. Bisher gibt es jedoch kaum medizindidaktische Empfehlungen zur Lehrgestaltung im Medizinstudium in diesem jungen Fachgebiet.

**Projektbeschreibung:**

Es wurde eine narrative Literaturübersicht zu medizindidaktischer Forschung im Fachgebiet Alterspsychiatrie erstellt. Weiterhin wurden bestehende Evaluationsdaten eines klinischen Seminars im Fachgebiet Alterspsychiatrie und -psychotherapie ausgewertet und eine Zielgruppenanalyse durchgeführt. Darauf basierend wurde in mehrdimensionalen Evaluationszyklen ein neues medizindidaktisches Konzept entwickelt, implementiert und evaluiert. Lernmaterial wurde über das Open-Source-Learning Management System ILIAS zur Verfügung gestellt.

**Ergebnisse:**

Insgesamt wurden 29 medizindidaktische Artikel und Projektberichte zum Fachgebiet Alterspsychiatrie identifiziert und ausgewertet. In der bestehenden Form wurde das Seminar zur Alterspsychiatrie an unserer Universitätsklinik bisher unterdurchschnittlich bewertet (Likert-Item Gesamtbewertung 4.3 von 6 im Vergleich zu anderen Seminaren mit einer durchschnittlichen Gesamtbewertung von 5.2, p<0.001). Das Seminar wurde evidenz-orientiert in Bezug auf Inhalt und Rhythmisierung überarbeitet. Für die Lernendenaktivierung wurden in der Vortragssequenz der Selbstreferenz-Effekt und Publikumsbefragung genutzt. Weiterhin wurden zwei gerontopsychiatrische Fallszenarien für die subjektive Auseinandersetzung adaptiert und für eine Plenumsdiskussion genutzt. Die Evaluationsergebnisse zeigten nach der Überarbeitung eine kontinuierliche Verbesserung und das Seminar wurde zuletzt mit 5.3 von 6 besser gesamtbewertet (p<0.01, U=135.5 Cohen’s d=1.28).

**Schlussfolgerung:**

Ein systematisches Vorgehen mit an medizindidaktischer Evidenz orientierten Entwicklung eines Seminarkonzeptes für Medizinstudierende war in unserem Kontext mit besseren Evaluationsergebnissen assoziiert. Die entwickelten Lehrmaterialien könnten lokal adaptiert für die alterspsychiatrische Lehre eingesetzt und entsprechend für andere Standorte und in Bezug auf Lernzuwachs evaluiert werden.

## Introduction

The field of geriatric psychiatry is comparatively young. According to the World Health Organization (WHO) projected demographic trends, it is expected that by 2050 about 20% of the population will be over 65 years of age, and 15% of this age group will have psychiatric disorders [1]. According to surveys by the Swiss Federal Statistical Office, the proportion of the population over 65 years of age is expected to increase the most in Switzerland [2]. Corresponding predictions for 2050 show a doubling of the over 80-year-olds to more than one million persons in Switzerland. From an epidemiological point of view, neurocognitive disorders, delirium, and depression are among the most common psychiatric disorders in old age [3], [4], [5].

Surprisingly, there is a disproportionately low rate of psychiatric treatment considering the higher rate of suicide among older adults [6]. Possible reasons are barriers to care, due to double stigmatization (age and mental illness), insufficiently qualified personnel, and lack of care structures [7], [8], [9]. The diagnosis and treatment of old-age psychiatric patients requires specific abilities, skills, and attitudes to adequately classify psychiatric symptoms (e.g. major depression in old age), manage behavioral disturbances (e.g. due to neurocognitive disorders), navigate the complex clinical situations, involving multimorbidity and polypharmacy, as well as master communication and specific developmental tasks and conflicts in old age [10], [11].

Despite the changing demographics and the required competencies for geriatric psychiatry care, there are few professorships in gerontological psychiatry in German-speaking countries, only one chair in gerontological psychiatry [12], and hardly any medical education recommendations for corresponding graduate training. Due to the changing demographics alone, geriatric psychiatry should, in principle, become increasingly important in graduate medical training. However, its position in the medical school curriculum still does not reflect this.

High-quality education and training of medical staff, and other health professionals is needed to close the geriatric psychiatry care gap. For example, medical students often do not rotate through geriatric psychiatric wards [13], [14]. Thus, clinical teaching in geriatric psychiatry, if taught at all, remains limited to lectures during undergraduate medical training. 

The aim of this educational case report was to review medical school geriatric psychiatry medical education literature and to use it for the concept development of a clinical seminar during a clerkship rotation. For this purpose, a narrative literature review was conducted (subproject 1), an assessment of seminar evaluations of geriatric psychiatry teaching in geriatric psychiatry rotations at the University Hospital for Geriatric Psychiatry and Psychotherapy (UPD Bern) was carried out (subproject 2), an evidence-oriented new seminar concept was developed (subproject 3), and a multidimensional pilot evaluation was conducted (subproject 4). 

## Project description

### Narrative literature review

The electronic database PubMed was searched using the following search terms and Boolean operators: (“geriatric psychiatry” AND “medical education” AND “students”). Furthermore, the individual digital archives of the journals “Academic Medicine”, “Medical Education”, “Medical Teacher”, “Academic Psychiatry” were searched with the search term (“geriatric psychiatry”) and “Der Nervenarzt” with the term (“teaching”). The term (“geriatric psychiatry”) was used for the search in the medical education database “MedEdPortal”. All identified articles were characterized and the educational focus and main findings were extracted (see attachment 1 ). The findings from the literature review were used to identify potential for instructional improvement. Changes in seminar evaluations were explored statistically in the context of a single academic context.

#### Assessment of student evaluations and context 

Available evaluation data of the seminar “Mental Illness in Aging” from the 2019 geriatric psychiatry clinical rotation (n=83 students) were analyzed thematically and compared to other psychiatric rotations during 2019: “Psychopathology”, “Sleep Disorders”, and “Psychiatric Interventions” (Wilcoxon signed-rank test for related samples, as the same students evaluated the different seminars). The clinical rotation begins in the 4th (six-year program) as part of the master’s degree in human medicine. Students rotate for one month (at full time) in internal medicine, surgery, gynecology, pediatrics, and psychiatry. The clinical rotation curricula are based on Entrustable Professional Activities (EPAs), which are defined in the PROFILES national learning objectives catalog [15].

For the target group analysis, a written survey of the perceived pros and cons of geriatric psychiatry was conducted (collected from n=21 students) and comments were sorted thematically. Student demographics, a detailed description of our teaching context, and the embedding of clinical rotation in psychiatry have already been published as part of other studies [16], [17].

#### Revision of the instructional design of the seminar

The learning objectives for the clinical rotation are based on the learning objectives cataloged in PROFILES [15]. For the revision of the seminar concept, a target group analysis was first conducted. Additional information was obtained from publications on geriatric psychiatry teaching, general medical education literature and current teaching recommendations of the University of Bern were also used [18]. Finally, expert advice from an instructional design course (“Best of Frontalunterricht” - Zentrum für universitäre Weiterbildung ZUW Hochschuldidaktik & Lehrentwicklung, Universität Bern) was obtained for optimising instructinal elements of the seminar [19]. All materials and geriatric psychiatry textbook chapters were made available to students on the digital learning platform “ILIAS” for self-regulated learning following the seminar.

#### Multidimensional pilot evaluation

After revising the seminar concept and piloting it for three months in the 2021 geriatric psychiatry clinical rotation (n=27 students, 6-point Likert item on global assessment), global assessment was evaluated in comparison to previous seminar evaluations from 2019 (clerkship seminar evaluations from eight months, n=83 students) (Wilcoxon rank-sum test, unconnected sample because students were from different clerkship cohorts). Effect size was calculated using Cohen's d for nonparametric tests [20]. For two implementations of the revised clinical rotations, an additional written multidimensional evaluation (5-point Likert items (n=15 items), plus three open-ended questions) was conducted on learning climate, lecturing aspects, and media use, among others. The questions of the evaluation form are listed in table 1 (Tab. 1) and in attachment 2 .

## Results

### Narrative literature review

A total of 659 articles and 15 MedEdPORTAL resources were found. Of these, 29 articles and 9 teaching project reports related to geriatric psychiatry teaching in medical school were retained. Table S1 (see attachment 1 ) shows the data extraction. The search term combination (“psychiatry” AND “GMS Journal for Medical Education”) yielded no hits. Most articles referred to geriatric psychiatry teaching in medical school in the United States and Canada (77%) and about one-fifth (23%) referred to the European educational context (Germany, UK, Portugal).

Thematically, the articles most frequently reported on geriatric psychiatry clinical teaching (e.g., clinical rotation curricula or participation in mobile geriatric psychiatric care in 21% of the articles) [21], [22], [23], [24], [25], [26], followed by specific teaching methods (e.g., imagination exercise or immersion simulation) for geriatric psychiatry subject areas (also in 21% of articles) [27], [28], [29], [30], [31], [32], teaching methods (e.g., courses or seminars) [33], [34], [35], [36], learning objectives for medical students [27], [37], [38] and geriatric psychiatry teaching needs (each in 14% of articles) [12], [13], [14], [39]. Individual articles reported on problem-based curricula in the field of psychiatry related to life stages [40], digital geriatric psychiatry teaching and learning resources [41], specific examination formats for medical students’ competencies in geriatric psychiatry [42], [43], [44] and career path decisions of medical students in relation to geriatric psychiatry [45].

For empirical studies, scales measuring prejudice against geriatric psychiatry patients and interest in a professional career in geriatric psychiatry were frequently used [23], [24], [29], [36]. Scales were found to measure the acquisition of knowledge, skills [22], [26], [33] and prejudices of medical students [26], [29], with predominantly significant improvements after substantial clinical exposure. In one controlled-randomized trial however, no change in career decision-making was observed [26]. 

Furthermore, evaluated English-language teaching materials were identified for the following geriatric psychiatric topics: differential diagnostic considerations for dementia, depression, and delirium [46]; Old age addiction [47]; Recognizing psychological and behavioral disturbances of dementia [48]; falls and fall prevention [49]; introduction to geriatric psychiatry [50]; cross-cultural communication strategies with older adults [51]; recognition and treatment of depression in the elderly [52]; and unintentional weight loss in old age [53]. An overview of the didactic methods used is shown in Table S1 (see attachment 1 ).

#### Assessment of the status of student evaluations

Analysis of the 2019 geriatric psychiatry clerkship seminars (data available from n=83 students, proportion female=59%, proportion male 41%, corresponding to an evaluation response of 78%, Likert-item overall rating average 4.3 out of 6, SD=0.9, median=4.3, skewness=-0.47) compared to other seminars with an average overall rating of 5.2 out of 6 (SD=0.8, median=5.3, skewness=-0.23, W=97, p<0.001) revealed evidence of a poorer average overall rating of the geriatric psychiatry seminar. The other clerkship seminars received lowest individual ratings ranging from 5.1 (SD=0.7), for example, for the psychopathology seminar to a maximum of 5.6 (SD=0.5) for the psychiatry clerkship introduction seminar. The overall evaluation of the clinical rotation on a 5-point Likert-item was 4.4 (SD=0.5).

No specific evaluations were available from the 2020 clinical rotations due to the COVID-19-pandemic.

In the target group analysis medical students without clinical exposure to geriatric psychiatry most frequently mentioned diversity of geriatric psychiatric patients (3/21), exposure to life experience (3/21), and societal relevance (3/21) as presumed positive aspects related to working in the field of geriatric psychiatry. In contrast, an expected one-sidedness of clinical activity (5/21), challenges in communicating with geriatric psychiatric patients (5/21), and a generally poorer prognosis (5/21) were classified as unattractive aspects about the specialty. The complexity of geriatric psychiatric patients was rated as both positive and unattractive (3/21 each). Of the students surveyed, the majority (62%) indicated they were still undecided about a specialty. 

#### Revision of the seminar concept

The seminar duration was about 60 minutes. Regarding the instructional design we considered research showing that the attention span dropping after around 25 to 30 minutes [54], theories on the influence of subjective theories, individually different prior knowledge, learning strategies, and learning motivation on the individual learning speed [55]. Three overarching learning objectives with reference to the General Objectives for the Medical Expert and Professional and to EPAs 3, 4 and 7 from PROFILES were formulated for the seminar content design [15]. In addition, ad hoc questions from the students were collected prior to each seminar.

For the cognitive activation during the lecture component of the seminar, the self-reference effect [56] was used at the beginning (imagining one's own aging), followed by a theory input on demographic development, the need for geriatric psychiatric care, and an overview of geriatric psychiatric syndromes and their treatment with interspersed clinical audience questions. Content was also selected in consideration of target group analysis. Primarily visualized overview graphics were used for the necessary condensation of information.

For the second part of the seminar, a work assignment in small groups (2-3 students) (see attachment 3 and attachment 4 ) was given of two short typical geriatric psychiatric case vignettes (major depressive episode and paranoid syndromes in neurocognitive disorder). Both case vignettes included questions about differential diagnoses and treatment recommendations, which were discussed afterwards. 

#### Multidimensional pilot evaluation

The iteratively adjusted geriatric psychiatry seminar was rated 5.3 out of 6 (SD=0.8, median=5) in February through April 2021 (electronic evaluation response global rating: 67%, data included from corresponding n=18 clerkship students, proportion female=61%, proportion male=39%), with an average of 5.3 out of 6 (SD=0.8, median=5.0, skewness=-0.19) evaluated better compared to the average of the geriatric psychiatry seminar global assessments from the 2019 cohort (n=83 students, proportion female=59%, proportion male 41%, overall average rating 4.3 out of 6, SD=0.9, median=4.3, skewness=-0.47) (p<0.01, U=135.5, Cohen's d for non-parametric tests=1.28). Results of the additional written multidimensional geriatric psychiatry seminar evaluation (n of two revised seminar evaluations=21 clerkship students and elective year students) are summarized in table 1 (Tab. 1). 

## Discussion

In the present work, we examined what evidence for instructional design is, to date, available for undergraduate teaching in the field of geriatric psychiatry. In addition, evidence-based general educational recommendations (including target group analysis, sequencing, and learner activation) were used to revise the instructional design of a geriatric psychiatry seminar in the clerkship year, that had previously been evaluated below average. The new seminar concept was evaluated significantly better by students. In this respect, the pilot evaluations presented here provide a first concrete indication that evidence-based improvements in instructional design can be statistically objectified by means of standardized evaluations.

In summary, the international literature on medical education shows that geriatric psychiatry instruction, its outcome assessment, and specific workplace-based teaching tend to be underrepresented in medical schools and do not adequately reflect societal needs [13], [22], [41]. Whereas previous studies [21], [28], [37] primarily examined geriatric psychiatry learning objectives and appropriate curricular structures, more recent research on geriatric psychiatry teaching has focused on the necessary human resource infrastructure [14], recruitment of junior faculty [26], digital teaching in geriatric psychiatry [41] and teaching methods for specific disease entities such as neurocognitive disorders [32].

Lack of geriatric psychiatry clinical rotation experience in medical education may make it difficult for students to relate to everyday clinical practice and sabotage the necessary constructive alignment regarding competency-based learning objectives, theory and clinical practice, and their assessment [57]. The quality of a workplace-based clinical learning experience in geriatric psychiatry care plays an important role for the individual acquisition of competencies [22]. Educational research regarding professional interest in the specialty of geriatric psychiatry seems most likely to be inconclusive due to curricular structure differences [25], [26]. In contrast, a meta-analysis of career interest in the specialty of general practice clearly concludes that early and systematic clinical learning experiences are associated with higher career interest in the specialty [58].

The prejudices and stereotypes mentioned by students (e.g., treatment options and outcomes) regarding geriatric psychiatry patients at our university hospital differ surprisingly little from those reported as early as the 1980s in the United States [21]. Therefore, in addition to subject-relevant preclinical teaching, clinical exposure, structured discussion of geriatric psychiatry clinical cases and of the diversity of geriatric psychiatry patients, seem essential for the reduction of prejudices and high-quality care [26], [29]. Clinical rotations in geriatric psychiatry also offer students the opportunity to engage with a conscious attitude towards age and aging from a bio-psycho-social perspective under appropriate supervision and to use this for future clinical work, regardless of specialty.

Seminars as reported in this pilot project should be seen as one element for teaching geriatric psychiatry and have to be complemented by theory and clinical experience. From a medical education point of view, it seems important to investigate the actual learning outcomes and attitude changes in an intervention-dependent manner. There is emerging evidence on the relevance of clinical rotations for changes in attitude, and career interest in this field in the context of medical students [22], [25] as well as residents [45], [59]. Accordingly, the various examination formats (knowledge tests, objective structured clinical examinations, and workplace-based assessments) should adequately reflect geriatric psychiatry learning objectives, in order to be able to assess the acquisition of competencies at higher levels.

## Conclusion

To adequately prepare medical students for their clinical practice and societal care needs, high-quality teaching formats, including clinical rotations, must be implemented for geriatric psychiatry. Some resources for evidence-based teaching in geriatric psychiatry are already available and can be used by educators, such as the materials examined in this project report.

## Note

If interested in materials beyond the supplementary materials, the authors of this manuscript can be contacted.

## Competing interests

The authors declare that they have no competing interests. 

## Supplementary Material

Table S1 Medical education articles

Evaluation sheet

Gereontopsychiatric case vignette 1

Gereontopsychiatric case vignette 2

## Figures and Tables

**Table 1:**
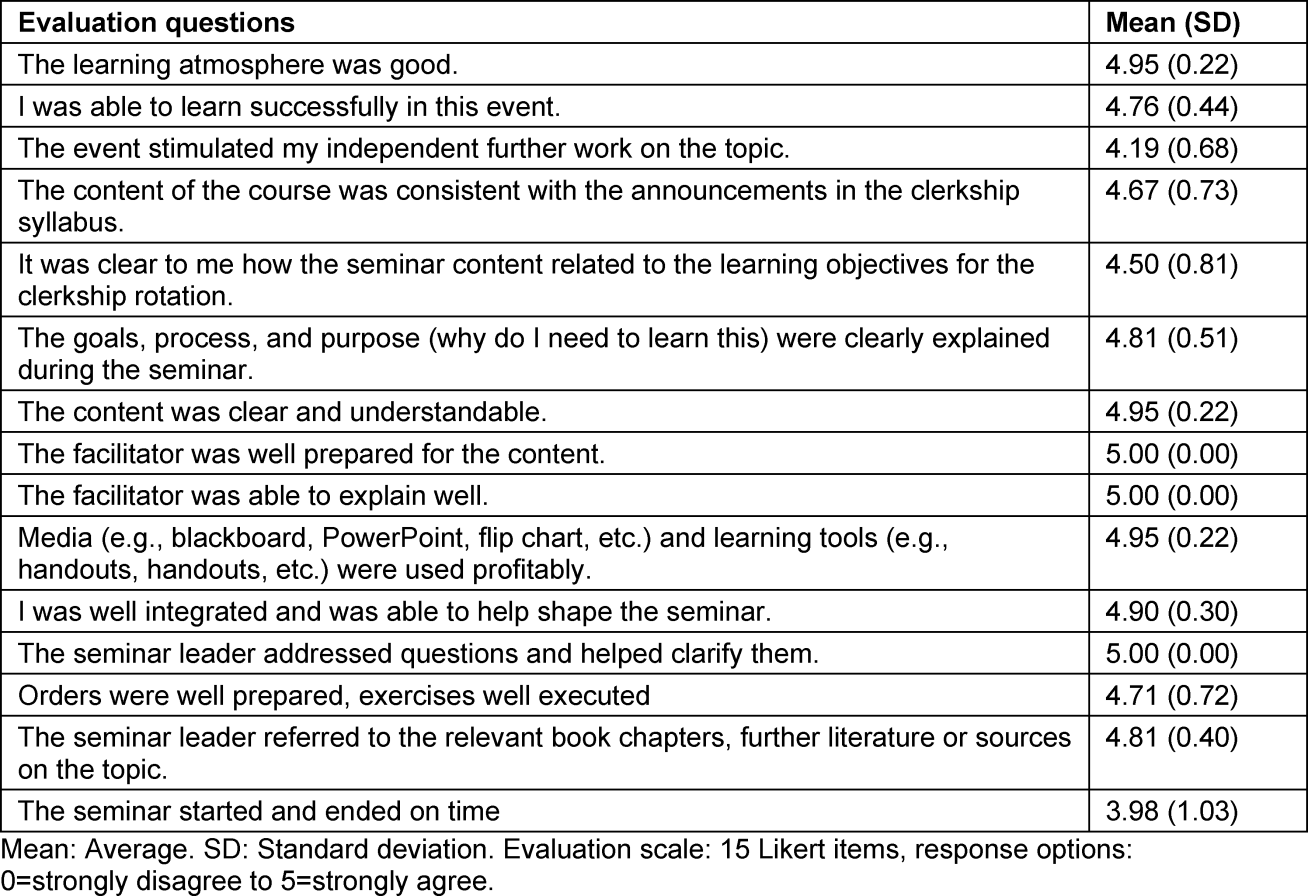
Multidimensional evaluation of the revised seminar
