# Supplementary material for: Evidence-oriented teaching of geriatric psychiatry: a narrative literature synthesis and pilot evaluation of a clerkship seminar
Source: GMS J Med Educ. 2022 Apr 14;39(2):Doc20. doi: 10.3205/zma001541 (PMC9174071; doi:10.3205/zma001541)
Supplement: Table S1 Medical education articles [file JME-39-20-s-001.pdf]

Attachment 1: Table S1 Medical education articles

Year / Country	Author	Focus	Content	Main result
1983 / USA	Molinari et al.	Clinical teaching	Curriculum development for a geriatric psychiatry block internship/family placement in the outpatient sector.	8-week elective clinical rotation with geriatric psychiatry curriculum.
1988 / USA	Marin et al.	Learning objectives	Learning objectives development and methodological overview of gerontological psychiatry teaching in medical school.	Curriculum topic blocks for students: Normal aging, psychopathology in aging and its clinical assessment, clinical block practicum, in-depth elective rotations.
1989 / UK	Isaacs et al.	Teaching method	Imagination exercise for students to develop empathy towards gerontological psychiatric patients	Didactic guide for 90-minute teaching unit. Good evaluation results according to authors, no quantitative data published.
1998 / Germany	Nicholas	Teaching needs	Survey study on geriatric and gerontopsychiatric teaching in Germany.	Need for specialized chairs to systematically embed geriatric psychiatry teaching.
1999 / USA	Goldstein et al.	Instruction (Course)	Curriculum evaluation of a 1-week dementia course for medical students.	Self-assessment of diagnostic competence higher than in control group.
2005 / USA	Halpain et al.	Instruction (Course)	Project report of a 10-week research course for medical students and residents in geriatric psychiatry.	85 applications, 35% selection rate, positive evaluation results (3.8 on 4-point Likert item).
2006 / USA	McFarland et al.	Instruction (Seminar)	Qualitative evaluation study of a seminar on communication skills by means of a structured life review for gerontopsychiatric patients	Medical students were able to deepen their understanding of connections between psychosocial, cultural, or spiritual life events, among others, and mental health.
2007 / Germany	Schäfer et al.	Teaching organization (studies)	Curricula overview of psychiatric topics in the reform course of studies in medicine of the Charité - Universitätsmedizin Berlin	Development of a teaching model based on problem-based learning, structured by. Organ systems and life stages, clinical exposure via block practicals and examination via multiple choice and objective structured clinical examinations.

2008 / USA	Zisook et al.	Learning objectives	Expert opinion on psychopharmacological teaching according to psychiatric subspecialties.	Low interest and motivation among students regarding psychopharmacology in old age psychiatry, underestimation of age-specific changes in pharmacodynamics and kinetics, partly insufficient data as a challenge for teaching.
2010 / Canada	Diachung et al.	Clinical teaching	Randomized-controlled trial of knowledge and skill acquisition by medical students in geriatrics and gerontopsychiatry.	Students with clinical rotations in geriatrics and geriatric psychiatry performed significantly better on knowledge exams and clinical-practice exams than the control group.
2010 / USA	Karel et al.	Testing	Development of an examination instrument for gerontological psychological competence	In survey evaluation, the instrument was rated as useful in terms of content, but impractical in terms of duration.
2010 / Portugal	Ribeiro et al.	Teaching needs	Review article on research and teaching in gerontological psychology and psychiatry in Portugal.	Increasing need for specific training of junior staff and corresponding education and training curricula.
2012 / Canada	Diachung et al.	Testing	Development of a clinical performance measurement tool for geriatric and gerontopsychiatric clinical examination of students.	High interrater reliability (90.4%), for 30/36 observation items over 80% agreement with gold standard.
2012 / USA	Roane et al.	Clinical teaching	Cohort study to measure attitudinal change in medical students after participation in an outreach clinical block clerkship (mobile geriatric psychiatry).	Positive evaluation results from students. No statistically significant difference in Maxwell-Sullivan Attitude Scale.
2013 / Canada	Haque et al.	Instruction (Seminar)	Attitudinal changes in medical students after attending a day-long seminar on clinical skills in geriatrics and geriatric psychiatry.	No significant difference before and after the day seminar in terms of UCLA Geriatrics Attitudes Scale.
2015 / USA	Curran et al.	Target group survey	Survey study of medical students' perspectives on academic careers in geriatric psychiatry.	Unawareness of career opportunities, potential financial loss, and stigma identified as barriers. Concrete clinical and research experience and need identified as positive factors.
2015 / USA	Lehmann et al.	Teaching needs	Survey study of geriatric psychiatry teaching programs at medical schools in the U.S.	21% of U.S. medical schools surveyed had no gerontological psychiatry course offerings.

2017 / USA	Lehmann et al.	Learning objectives	Expert consensus on geriatric psychiatry learning objectives for medical students.	6 content areas of focus: Normal aging, clinical diagnosis of mental health in the elderly, psychopharmacology in the elderly, depression, dementia, delirium.
2016 / USA	Blazek et al.	Clinical teaching	Attitude change toward dementia in students after participation in a two-day clinical teaching practicum in dementia homes.	No quantitative difference in attitudes towards dementia patients. In focus groups, students reported understanding how dementia care is delivered in a home through participation.
2016 / USA	Ray-Griffith et al.	Clinical teaching	Cohort study of interest in geriatric psychiatry among medical students as a function of clinical experience.	Only students with a clinical rotation in geriatric psychiatry indicated a statistically significant higher interest in pursuing a career in this specialty.
2017 / USA	Abreu et al.	Teaching method	Pre-/post-program evaluation of an immersion simulation on cognitive and sensory load in aging.	Statistically significant and positive attitudinal changes of students in the Approaches to Dementia Questionnaire.
2017 / Germany	Siegel et al.	Testing	Explorative study of the proportion of psychiatric and psychotherapeutic questions in the written part of the second state examination in medical studies.	Overall 6.5% share of psychiatric questions. Gerontopsychiatric issues (including organic mental disorders) also underrepresented.
2017 / USA	Wilkins et al.	Learning objectives, teaching methods	Expert consensus on gerontological psychiatry learning objectives and corresponding teaching methods in medical school.	Various teaching units on normal aging, clinical diagnosis of mental health in old age, psychopharmacology in old age, depression, dementia, delir available on MedEdPortal.
2019 / Germany	Baessler et al.	Teaching method	Randomized trial comparing the effectiveness of different teaching methods on delirium.	Video instructional materials alone or in combination with instructional text were signficantly better than instructional text alone for knowledge acquisition.
2019 / Canada	Chun et al.	Teaching needs	Survey study of geriatric psychiatry teaching in medical schools in Canada.	Heterogeneous gerontological psychiatry teaching in Canadian medical schools.
2019 / Canada	Pokrzywko et al.	Clinical teaching	Randomized-controlled study of medical students' interest in gerontological psychiatry careers dependent on clinical rotations.	Significantly higher comfort in dealing with gerontological psychiatric patients* with clinical rotation in gerontological psychiatry. No difference in interest in working in gerontological psychiatry.

2020 / USA	Blazek et al.	Teaching material	Review paper on digital teaching/learning resources in geriatric psychiatry.	Published bibliography at www.admsep.org/ geriatricbibliography.php
2021 / Germany	Besse et al.	Teaching method	Program evaluation of psychiatric teaching after conversion to digital teaching formats at Göttingen Medical Faculty	In multidimensional self-assessments of medical students, no differences in subjective learning gains (including "dementia" domain).
2021 / USA	Hernandez et al.	Teaching method	Review paper (scoping review) on didactic methods in the field of neurocognitive disorders.	Research gap in teaching neurocognitive disorders to medical students and psychiatric residents*.

Evaluated medical didactic resources for teaching geriatric psychiatry (MedEdPORTAL, last updated search April 26, 2021):

**Year / Country**	**Author**	**Teaching topic**	**Didactic methods**
2006 / USA	Paniagua et al.	Differential diagnosis between dementia, depression and delirium	Small group activity with card sorting and facilitated group discussion.
2012 / USA	Bee Field	Substance abuse in old age	Keynote lecture, knowledge test, reinforcement material for a self-directed learning module for students and residents.
2013 / USA	Rockefeller et al.	Recognizing mental and behavioral symptoms of dementia	Workshop for medical students and residents with keynote lecture, discussion of clinical work material, and case-based application exercise.
2014 / USA	Cheng	Fall and fall prevention	Team-based learning activity for medical students with preparation material, individual and group-based work assignments, knowledge review and discussion of results.
2014 /USA	Blazek	Introduction Allterspsychiatry	Seminar for medical students, keynote lecture, knowledge assessment, case work in small groups and discussion of results.
2016 / USA	Agness-Whittaker et al.	Culturally competent health communication with older patients	Seminar for medical students and residents with preparation material, impulse lecture, self-reflection and role play.
2017 / USA	Lehmann et al.	Diagnosis and treatment of depressive disorders in old age	Two digital, self-paced learning modules for medical students with video lectures, clinical teaching videos, and knowledge tests.
2017 / USA	Mulligan et al.	Diagnosis and treatment of unintentional weight loss in geriatric patients	Multi-part curriculum with keynote lectures, role-playing with drama patients, case work, and application exercises for clinical examination and treatment planning.
