# Supplementary material for: Evidence-oriented teaching of geriatric psychiatry: a narrative literature synthesis and pilot evaluation of a clerkship seminar
Source: GMS J Med Educ. 2022 Apr 14;39(2):Doc20. doi: 10.3205/zma001541 (PMC9174071; doi:10.3205/zma001541)
Supplement: Evaluation sheet [file JME-39-20-s-002.pdf]

Attachment 2: Evaluation sheet

UNIVERSITÄRE PSYCHIATRISCHE DIENSTE BERN

UNIVERSITÄT BERN

Seminar Evaluation Psychiatry Clerkship

Seminar Topic: **Geriatric Psychiatry and psychotherapy.**
Date: **XY**
Seminar leader: **Dr. med. XY**

Please mark your rating in each case.

	0 = does not apply at all<br>5 = fully applies					
The learning atmosphere was good.	0	1	2	3	4	5
I was able to learn successfully in this event.	0	1	2	3	4	5
The event stimulated my independent further work on the topic.	0	1	2	3	4	5
The content of the course was consistent with the announcements in the clerkship syllabus.	0	1	2	3	4	5
It was clear to me how the seminar content related to the learning objectives for the clerkship rotation.	0	1	2	3	4	5
The goals, process, and purpose (why do I need to learn this) were clearly explained during the seminar.	0	1	2	3	4	5
The content was clear and understandable.	0	1	2	3	4	5
The facilitator was well prepared for the content.	0	1	2	3	4	5
The facilitator was able to explain well.	0	1	2	3	4	5
Media (e.g., blackboard, PowerPoint, flip chart, etc.) and learning tools (e.g., handouts, handouts, etc.) were used profitably.	0	1	2	3	4	5
I was well integrated and was able to help shape the seminar.	0	1	2	3	4	5
The seminar leader addressed questions and helped clarify them.	0	1	2	3	4	5
Orders were well prepared, exercises well executed	0	1	2	3	4	5
The seminar leader referred to the relevant book chapters, further literature or sources on the topic.	0	1	2	3	4	5
The seminar started and ended on time	0	1	2	3	4	5

Please note the reverse side!

Attachment 2 to Lenouvel E, Lornsen F, Schüpbach B, Mattson J, Klöppel S, Pinilla S. *Evidenz-oriented teaching of geriatric psychiatry: A narrative literature synthesis and pilot evaluation of a clerkship seminar.* GMS J Med Educ. 2022;39(2):Doc20. DOI: 10.3205/zma001541

What was particularly good about the seminar?
(e.g. presentation of contents, working methods, methods, etc.)

What else would have been desirable?
(e.g. presentation of contents, working methods, methods, etc.)

Other comments or suggestions?

Thank you very much!

Attachment 2 to Lenouvel E, Lornsen F, Schüpbach B, Mattson J, Klöppel S, Pinilla S. *Evidenz-oriented teaching of geriatric psychiatry: A narrative literature synthesis and pilot evaluation of a clerkship seminar.* GMS J Med Educ. 2022;39(2):Doc20. DOI: 10.3205/zma001541
