# Supplementary material for: Evidence-oriented teaching of geriatric psychiatry: a narrative literature synthesis and pilot evaluation of a clerkship seminar
Source: GMS J Med Educ. 2022 Apr 14;39(2):Doc20. doi: 10.3205/zma001541 (PMC9174071; doi:10.3205/zma001541)
Supplement: Gereontopsychiatric case vignette 1 [file JME-39-20-s-003.pdf]

**Attachment 3: Gereontopsychiatric case vignette 1**

# Mr. Beat W.

Clinical rotation geriatric psychiatry – case study

**Patient Information**:

Beat W. is an 85-year-old, married, white male, with no psychiatric history. Six months after an eye operation due to a cataract, he was brought to the emergency room after cuts on both wrists and a geriatric psychiatric consultation was requested.

**Main complaint:**

“I’m not depressed. I want to go home.”

**History**:

Mr. W. was brought to the emergency room by the medical police with deep cuts on both wrists. His wife had returned unexpectedly early from shopping, having forgotten her cell phone at home. She found Mr. W. bleeding in the bathroom and called the ambulance service.

In the emergency room, the cuts were treated and a geriatric psychiatric consultation was requested: “From a medical point of view, Mr. W. can go home. His blood pressure and diabetes are well controlled. We need an geriatric psychiatric evaluation because it was a suicide attempt. Currently, he denies suicidal ideation.”

Mr. W. states that he is not sad and can take care of himself. He is independent and does not need any other help at home. He did not want to take any sedatives and wanted to be left alone.

Mr. W.’s wife reports that he has not been the same since the eye operation. He had previously enjoyed walking in the forest, working in his home workshop and making wooden figures. He read the newspaper daily and was interested in politicals. Recently, he had often sat alone in a dark room, talking little and no longer watching the news. He was having trouble falling asleep, waking up frequently, and had little appetite. She had reported this to their family doctor, who had prescribed sertraline. Mr. W. had taken this for a few days, said “it doesn't help at all” and stopped taking it again. He had always been a stoic type, did not like to complain and hated medication.

Attachment 3 to Lenouvel E, Lornsen F, Schüpbach B, Mattson J, Klöppel S, Pinilla S. *Evidenz-oriented teaching of geriatric psychiatry: A narrative literature synthesis and pilot evaluation of a clerkship seminar.* GMS J Med Educ. 2022;39(2):Doc20. DOI: 10.3205/zma001541

Mr. W. was seen by a doctor in a pain consultation the week before due to chronic severe back pain. A trial of gabapentin was started but “did not help at all” and was discontinued by Mr. W. after a few days.

**Questions:**

1. What additional history would you collect?
2. Would you still ask specific questions? Which ones?

**Mental Status Exam:**

Awake, personal hygiene slightly neglected, otherwise age-appropriate appearance, little eye contact. Slight response latencies, monosyllabic, otherwise coherent in formal thought. No evidence of thought disturbances in content. Mood was “not sad,” reduced vibratory capacity, mildly depressed-like affect. Suicidal and other-aggressive thoughts or plans are denied.

Mini-Mental Status MMS: 27/30, deductions for name of hospital, 1/5 incorrect for spelling backwards, and for “No ifs, ands, or buts.”

**Labs:**

All routine tests unremarkable, including blood count, liver function values, thyroid values, and urinalysis.

**Cerebral Imaging**:

No evidence of acute processes on MRI. No atrophy, ventricular width unremarkable, medullary hyperintensities (mild severity) compatible with chronic cerebrovascular disease.

**Questions:**

1. What is your main suspected diagnosis?
2. How would you assess the risk of suicide?
3. What treatment setting do you recommend?
4. What treatment do you recommend?
