# Supplementary material for: Evidence-oriented teaching of geriatric psychiatry: a narrative literature synthesis and pilot evaluation of a clerkship seminar
Source: GMS J Med Educ. 2022 Apr 14;39(2):Doc20. doi: 10.3205/zma001541 (PMC9174071; doi:10.3205/zma001541)
Supplement: Gereontopsychiatric case vignette 2 [file JME-39-20-s-004.pdf]

Attachment 4: Gereontopsychiatric case vignette2

Ms Helen S. – For students

Clinical rotation geriatric psychiatry – case study

Reason for consultation

Helen S. is a 73-year-old divorced white woman who was referred by her primary care physician for evaluation of paranoid thinking (persons would invade her home and steal or hide things). Ms. S. had refused referral, so her primary care physician started olanzapine 2.5 mg and dosed up to 5 mg. After three weeks, she agreed to be referred.

Main complaint:

"My brother wants me to come here."

History:

Mrs. S. had lived alone for the last 3-5 years and had always drawn the curtains at home. She has little contact with her family, although she previously had a good relationship with them. She states that she has been somewhat sad since she retired 8 years ago. She had worked as a real estate agent. Furthermore, she was worried because of intruders in her apartment. She had never seen them, but if she did, she would not hurt them but would call the police. She had bought a pistol for her protection, but her brother had taken it from her. She had spent thousands of francs changing her door locks in the last few years.

She otherwise sleeps well, eats normally, has no sensory delusions, and has no suicidal or threatening thoughts or plans. She is not taking any other medications. She has had no significant medical conditions, has "always been healthy," and has never smoked. She had also never drunk alcohol or consumed other substances and had never received psychiatric treatment.

Social history:

Mrs. S. had three sons, but rarely had contact. One brother was very helpful. She had grown up on a farm in the Bernese Oberland, was "poor but happy" and had a secondary school diploma. She used to work as a stenographer for the Swiss army. She has an elderly neighbor who is a friend and goes shopping with her because she can't manage well on her own.

Attachment 4 to Lenouvel E, Lornsen F, Schüpbach B, Mattson J, Klöppel S, Pinilla S. *Evidenz-oriented teaching of geriatric psychiatry: A narrative literature synthesis and pilot evaluation of a clerkship seminar.* GMS J Med Educ. 2022;39(2):Doc20. DOI: 10.3205/zma001541

Questions: 1. What additional history would you collect?

Mental Status Exam:

Alert, friendly in contact, cooperative. Casually dressed and well-groomed, adequate for weather. Unobtrusive eye contact, rate of speech unobtrusive. Coherent in formal thought, purposeful. No evidence of auditory or visual hallucinations. Complex delusional paranoid experience (strangers in the home moving or hiding objects). Irritable mood when thematizing the strange persons in the apartment, otherwise joyful mood. Noticeably indifferent affect in relation to stressful situation. Overall, no indication of acute danger to self or others.

Cognitive testing:

Mini mental status exam: 19/30 points. Deficits in backward spelling (refused). Continued deficits in backward arithmetic and visuoconstruction. Remembered 2/3 objects after 3 minutes. Could name 10 animals in one minute, perseverated on "dog, cat and squirrel". Clock test: circle intact, numbers correctly placed, short and long hands reversed.

Physical Exam/Lab:

Physical examination, including vital signs unremarkable. All routine examinations unremarkable, including blood count, liver function values, thyroid values, vitamin B12 and urine status.

Cerebral imaging:

A CT scan 17 months ago revealed few focal hypodensities, consistent with ischemic microangiopathy.

Questions:

1. What is your diagnosis?
2. Which treatment setting do you recommend?
3. Which medication do you recommend?
4. How do you evaluate the use of antipsychotics in patients with dementia?
